# The oral–sleep interface: Associations between oral health status and sleep outcomes in Canadian adults

**DOI:** 10.1371/journal.pone.0350313

**Published:** 2026-10-21

**Authors:** Anas J. Mistareehi, Ibrahim Hoja, Wafaa Mohamoud, Jessica R.L. Lieffers, George S. Katselis

**Affiliations:** 1 Department of Anatomy, Faculty of Medicine, Jordan University of Science and Technology, Irbid, Jordan; 2 College of Pharmacy and Nutrition, University of Saskatchewan, Saskatoon, Saskatchewan, Canada; 3 Department of Medicine, Canadian Centre for Rural and Agricultural Health, College of Medicine, University of Saskatchewan, Saskatoon, Saskatchewan, Canada; Hamadan University of Medical Sciences School of Dentistry, IRAN, ISLAMIC REPUBLIC OF

## Abstract

**Background:**

Sleep disturbances and oral health problems are both prevalent public health concerns with substantial impacts on quality of life and systemic health. However, population-based evidence examining the independent associations between multiple dimensions of oral health and multiple sleep outcomes remains limited. This study investigated the associations between oral health indicators and sleep disturbances among Canadian adults using nationally representative survey data.

**Methods:**

We utilized data from adults aged 18 years and older who completed the 2017–2018 Canadian Community Health Survey (CCHS). Four oral health indicators were assessed: self-reported oral health, oral pain, bleeding gums, and dry mouth. Four sleep outcomes were examined: short sleep duration, insomnia symptoms, unrefreshing sleep, and difficulty staying awake. Multivariable logistic regression models were used, with progressive adjustment for sociodemographic factors, health status, and health behaviors.

**Results:**

All four oral health indicators were significantly associated with most sleep outcomes in the full adjusted model. For short sleep duration, the strongest association was observed for poor oral health (AOR: 1.623). For insomnia symptoms, dry mouth (AOR: 1.904) and oral pain (AOR: 1.826) exhibited the strongest associations. For unrefreshing sleep, oral pain (AOR: 1.937) and dry mouth (AOR: 1.808) were associated with higher odds of reporting unrefreshing sleep. For difficulty staying awake, dry mouth (AOR: 1.720) and oral pain (AOR: 1.701) showed the strongest associations.

**Conclusion:**

Oral health problems are associated with multiple dimensions of sleep disturbance. These findings highlight the need for integrated care and complementary screening between dental and sleep medicine.

## Introduction

Sleep is a fundamental biological process supporting metabolic, immune, neurocognitive, and emotional function, as well as quality of life [1]. Both insufficient and excessive sleep duration, along with poor sleep quality, are associated with adverse health outcomes including obesity, diabetes, cardiovascular disease, depression, impaired daytime function, and increased mortality [2,3]. Current evidence suggests that 7–8 hours of sleep per night is most favorable for adult health [4], while insomnia symptoms, unrefreshing sleep, and excessive daytime sleepiness may reflect broader physiologic dysregulation and chronic disease burden [3]. Sleep may also influence oral health through immune and inflammatory dysregulation, altered pain processing, endocrine disruption, behavioral changes, and reduced self-care motivation [5]. Emerging evidence suggests sleep disorders are an underrecognized risk factor for periodontal disease and other oral problems, reinforcing the close interconnection between sleep and oral health as components of general health [6,7].

Oral health is essential to overall well-being, with implications for nutrition, communication, social participation, pain, and systemic health [8]. Conditions such as gingival bleeding, chronic oral pain, and xerostomia can impair daily functioning and disturb sleep quantity and quality [6,9,10]. Oral pain can delay sleep onset, fragment sleep continuity, and intensify the reciprocal cycle between pain sensitization and sleep disruption [7]. Xerostomia has been linked to poorer sleep quality and greater daytime sleepiness through nocturnal discomfort and increased awakenings [10]. Similarly, gingival bleeding is intertwined with sleep through systemic inflammation; sleep deprivation increases the risk of gum inflammation, which in turn can perpetuate sleep disruption, reinforcing a cycle that links oral and systemic health [9,11]. In parallel, adults with sleep disorders are more likely to report dental pain, periodontal problems, and oral health related functional limitations [12]. Reviews further suggest that short sleep, poor sleep quality, insomnia symptoms, and sleep disordered breathing may contribute to gingival and periodontal inflammation via systemic inflammatory and behavioral pathways [6]. Together, these findings support consistent associations in which poor oral health may worsen sleep, while poor sleep may exacerbate oral disease risk and symptom burden.

Despite growing recognition of the oral sleep interface, population-based evidence remains limited, particularly studies examining multiple oral health and sleep outcomes simultaneously in nationally representative adult populations. This gap is significant in Canada, where oral health disparities and sleep problems are major public health concerns [13]. Using data from the 2017–2018 Canadian Community Health Survey (CCHS), this study examines whether oral health status, including self-reported oral health, bleeding gums, oral pain, and dry mouth, is associated with sleep quantity and quality, measured by sleep duration, insomnia symptoms, unrefreshing sleep, and difficulty staying awake. We hypothesize that poorer oral health and a higher burden of oral symptoms will be associated with unfavorable sleep outcomes, independent of sociodemographic, behavioral, and health-related factors. Accordingly, this study aims to examine these associations in Canadian adults to inform the integration of oral health into sleep medicine and public health frameworks.

## Methods

### Study design and participants

This cross-sectional study drew on data from the public use microdata file of the 2017–2018 CCHS. Conducted annually, the CCHS collects information on health status, health determinants, and patterns of healthcare utilization among non-institutionalized Canadians aged 12 years and older. The 2017–2018 survey cycle comprised 113,290 respondents, that includes a nationally representative sample across the ten provinces and three territories of Canada. The survey excluded individuals living on First Nations reserves or Aboriginal settlements, youth in foster care (aged 12–17 years), full-time members of the Canadian Armed Forces, residents of institutional facilities, and those residing in selected health regions of Quebec. Altogether, these exclusions accounted for less than 3% of the target population. Detailed descriptions of the sampling methodology and survey instruments have been published elsewhere [14,15]. For our analyses, we restricted the sample to respondents aged 18 years and older (n = 104,636). Descriptive analyses included participants with available data for the corresponding sleep outcome, whereas survey-weighted logistic regression analyses were restricted to participants with complete data for the corresponding sleep outcome, oral health indicator, and covariates included in each model. Consequently, the analytic sample size varied across outcomes and regression models because of missing data. The participant selection process is summarized in Fig 1. Because the data used were derived from publicly available microdata files, this study was exempt from research ethics board review.

**Fig 1:**
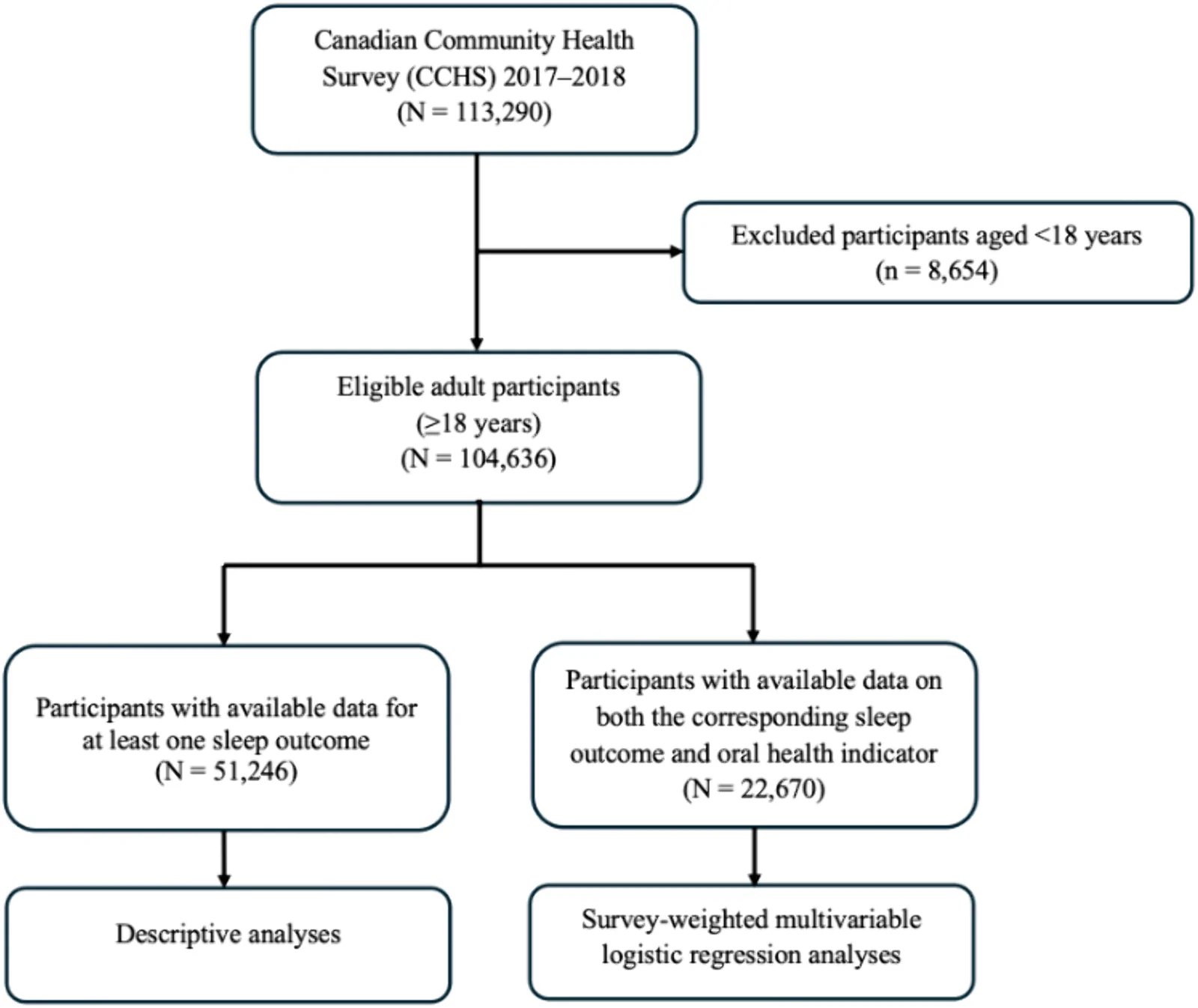
Flow diagram of participant selection from the 2017–2018 Canadian Community Health Survey (CCHS). Adults aged ≥18 years were eligible for inclusion. Descriptive analyses included participants with available data for each sleep outcome. For survey-weighted logistic regression analyses, participants were included if data were available for the sleep outcome and oral health indicator. Therefore, the analytic sample size varied slightly across sleep outcomes and regression models due to missing data.

### Variable definitions

#### Dependent variables: Sleep disturbances.

The outcomes of interest were self-reported sleep disturbance indicators, including short sleep duration, insomnia symptoms, unrefreshing sleep, and difficulty staying awake. Sleep duration was assessed by asking respondents: “How long do you usually spend sleeping each night?” with response options ranging from under 2 hours to 12 hours or more. For analytical purposes, we categorized sleep duration as adequate (≥7 hours) or inadequate sleep duration (<7 hours), consistent with previous literature [4]. Insomnia symptoms were assessed by asking: “How often do you have trouble going to sleep or staying asleep?” Response options included “never,” “rarely,” “sometimes,” “most of the time,” and “all of the time.” Respondents who answered either “most of the time” or “all of the time” were classified as having nighttime insomnia symptoms [16,17]. Unrefreshing sleep was assessed by asking: “How often do you find your sleep refreshing?” using five response options. Respondents who answered either “never” or “rarely” were considered to have unrefreshing sleep. Finally, difficulty staying awake was assessed by asking: “How often do you find it difficult to stay awake when you want to?” with the same previously five response options. Responses of “most of the time” and “all of the time” were combined to indicate difficulty staying awake [17].

### Primary independent variables

The oral health indicators were self-reported oral health measures, including overall oral health status, oral pain, bleeding gums, and dry mouth. The overall self-reported status of oral health was derived from the CCHS question: “In general, would you say your oral health?” Responses included “Excellent,” “Very Good,” “Good,” “Fair,” and “Poor.” We regrouped the responses into the following categories: “Poor” (fair/poor) and “Good” (excellent/very good/good) to create a binary exposure variable suitable for logistic regression analysis. The presence of specific oral health problems was derived from three separate questions. Oral pain was assessed by asking: “How often have you had any other persistent or ongoing pain anywhere in your mouth?” Bleeding gums were assessed by asking: “How often have you had bleeding gums, including while brushing or flossing your teeth?” Dry mouth was assessed by asking: “How often have you had persistent dry mouth?” For each question, response options were “Often,” “Sometimes,” “Rarely,” and “Never.” For analysis, responses were regrouped into the following categories: “Yes” (often/sometimes) and “No” (rarely/never) [13].

### Covariates

Sociodemographic characteristics: Self-reported sex was categorized as male or female. Age was grouped into the following categories: 18–34 years, 35–49 years, 50–64 years, and 65 years and older. Education level was classified as “less than secondary school graduation,” “secondary school graduation,” and “post-secondary certificate diploma or university degree.” Marital status was grouped into “Married/Common-law,” “Widowed/Divorced/Separated,” and “Single.” Income was categorized using six original response options (from “No income or income loss” to “$80,000 or more”) and regrouped into four categories: less than $20,000; $20,000–$59,999; $60,000–$79,999; and $80,000 or more. For health status, self-reported general health and self-rated mental health were derived from questions asking: “In general, would you say your general health is?” and “In general, would you say your mental health is?” with responses of “Excellent,” “Very Good,” “Good,” “Fair,” or “Poor.” These response options were dichotomized into “Poor” (fair/poor) and “Good” (excellent/very good/good). Body mass index (BMI) was calculated from self-reported height and weight and categorized as underweight (<18.5), normal weight (18.5–24.9), overweight (25–29.9), or obese class I, II, and III (≥30) [18]. For health behaviors: Alcohol consumption was categorized as regular drinker, occasional drinker, or no drinking in the past 12 months. Smoking status was regrouped into three categories: “Current daily/occasional smoker (current smoker),” “Former daily/occasional smoker (former smoker),” and “Experimental smoker or lifetime abstainer (never smoker).”

### Statistical analysis

Univariate logistic regression models were employed to evaluate the crude associations between covariate (sociodemographic, health status, and behavioral factors) with the four sleep disturbance outcomes (short sleep duration, insomnia symptoms, unrefreshing sleep, and difficulty staying awake). Results are reported as unadjusted odds ratios (ORs) with 95% confidence intervals (CIs). These analyses were conducted without applying survey weights and were performed using SPSS Statistics, Version 30.0 (IBM Corp., Armonk, NY, USA).

A series of survey-weighted multivariable logistic regression models were fitted to examine the independent associations between oral health indicators (poor oral health, oral pain, bleeding gums, and dry mouth) and sleep disturbance outcomes. Each oral health indicator was examined separately in a distinct regression model and was not mutually adjusted for the other oral health indicators. Model 1 examined crude (unadjusted) associations. Model 2 was adjusted for demographic characteristics (sex and age). Model 3 was adjusted for sex, age, education, BMI, smoking status, alcohol consumption, perceived general health, and perceived mental health. Model 4 represented the fully adjusted model and was further adjusted for income and marital status [11,19,20]. The same modeling approach was applied across all four sleep outcomes. All survey-weighted analyses incorporated the CCHS person-level sampling weight (WTS_M) and the 1000 bootstrap replicate weights to obtain nationally representative estimates and valid variance estimates that accounted for the complex multistage stratified survey design, in accordance with Statistics Canada recommendations. Weighted logistic regression models were fitted using the svyglm function in the survey package in R version 4.6.1 (R Foundation for Statistical Computing, Vienna, Austria). Model 1 results are presented as crude odds ratios (ORs), whereas the fully adjusted Model 4 results are presented in the main manuscript as adjusted odds ratios (AORs) with corresponding 95% confidence intervals (CIs). Results for Models 2 and 3 are provided in the Supplementary Material Table 1. To account for multiple testing across the 16 primary associations examined in the fully adjusted Model 4 (four oral health indicators × four sleep outcomes), p-values were adjusted using the Benjamini–Hochberg false discovery rate (FDR) procedure. Missing data were managed using an available-case approach; participants with complete data for the variables required in a given analysis were included, resulting in variable sample sizes across analyses. Multicollinearity among the independent variables included in the fully adjusted model (Model 4) was assessed using variance inflation factors (VIFs). All VIF values were below 5 (range: 1.03–1.46), indicating no evidence of problematic multicollinearity. Statistical significance was set at p < 0.05.

**Table 1:** Univariate logistic regression examining the associations between sociodemographic and behavioral characteristics with sleep (sleep duration, insomnia, unrefreshing sleep, and difficulty staying awake) among Canadian adults aged 18 years and older using data from the Canadian Community Health Survey (CCHS 2017–2018) Results are presented as odds ratios (ORs) with 95% confidence intervals (CIs). Statistical significance was set at p < 0.05. Ref = reference category, N = number of respondents.

	Sleep Duration					Insomnia			
	Adequate duration (≥7) (N)	Inadequate duration (<7) (N)	OR (95% CI)	P-Value		No(N)	Yes(N)	OR (95% CI)	P-Value
**Sex**									
Male	13167	10488	Ref			19935	3778	Ref	
Female	16194	11101	0.861 (0.831-0.891)	<0.001		20901	6521	1.646 (1.575-1.721)	<0.001
**Age Group (years)**									
18-34	6675	4637	Ref			9238	2110	Ref	
35-49	6231	4984	1.151 (1.092-1.214)	<0.001		8994	2248	1.094 (1.024-1.169)	0.008
50-64	7530	6195	1.184 (1.126-1.245)	<0.001		10579	3202	1.325 (1.246-1.409)	<0.001
≥65	8925	5773	0.931 (0.886-0.979)	0.005		12025	2739	0.997 (0.936-1.062)	0.932
**Marital status**									
Single	6972	5318	Ref			9689	2662	Ref	
Married/common law	16324	11365	0.913 (0.874-0.953)	<0.001		22663	5085	0.817 (0.775-0.861)	<0.001
Widowed/divorced/separated	6009	4855	1.059 (1.006-1.116)	0.030		8398	2528	1.096 (1.030-1.165)	0.004
**Income**									
Less than $20,000	399	301	Ref			537	165	Ref	
$20,000 to $59,999	16593	12039	0.968 (0.832-1.126)	0.670		22417	6236	0.905 (0.761-1.083)	0.270
$60,000 to $79,999	8352	6022	0.956 (0.820-1.115)	0.563		11825	2593	0.714 (0.598-0.856)	<0.001
$80,000 or more	4071	3195	1.040 (0.890-1.217)	0.621		5998	1286	0.698 (0.582-0.841)	<0.001
**Education**									
Less than secondary school	4115	3322	Ref			5791	1694	Ref	
Secondary school graduation	6316	5096	0.999 (0.942-1.060)	0.985		9007	2452	0.931 (0.868-0.998)	0.045
Post-secondary certificate/univ degree	18540	12871	0.860 (0.817-0.905)	<0.001		25502	5990	0.803 (0.755-0.853)	<0.001
**General Health**									
Good	26298	18109	Ref			36934	7600	Ref	
Poor	3034	3449	1.651 (1.567-1.740)	<0.001		3864	2678	3.368 (3.187-3.559)	<0.001
**Mental Health**									
Good	27631	19532	Ref			38835	8473	Ref	
Poor	1694	2011	1.679 (1.570-1.796)	<0.001		1946	1795	4.228 (3.948-4.527)	<0.001
**BMI**									
Normal Weight	12458	8277	Ref			16899	3895	Ref	
Underweight	588	424	1.085 (0.955-1.233)	0.210		773	243	1.364 (1.172-1.579)	<0.001
Overweight	9771	7290	1.123 (1.078-1.170)	<0.001		13872	3228	1.010 (0.959-1.063)	0.718
Obese—Class I, II, III	5536	4941	1.343 (1.281-1.409)	<0.001		7977	2543	1.383 (1.307-1.464)	<0.001
**Alcohol Drinking**									
No drinking in the last year	5191	4098	Ref			7453	1919	Ref	
Occasional	4262	3736	1.110 (1.046-1.179)	<0.001		6145	1875	1.185 (1.103-1.274)	<0.001
Regular	19802	13675	0.875 (0.835-0.916)	<0.001		27089	6463	0.927 (0.875-0.981)	0.009
**Smoking**									
Never	14853	10253	Ref			20866	4343	Ref	
Current	4947	4563	1.336 (1.274-1.401)	<0.001		7096	2459	1.665 (1.574-1.761)	<0.001
Former	9486	6717	1.026 (0.985-1.068)	0.214		12768	3472	1.306 (1.243-1.373)	<0.001
	**Unrefreshing Sleep**					**Difficult to stay awake**			
	No(N)	Yes(N)	OR (95% CI)	P-Value		No(N)	Yes(N)	OR (95% CI)	P-Value
**Sex**									
Male	20520	3123	Ref			22260	1366	Ref	
Female	22844	4546	1.309 (1.245-1.374)	<0.001		25521	1818	1.161 (1.080-1.248)	<0.001
**Age Group (years)**									
18-34	9556	1781	Ref			10542	781	Ref	
35-49	9268	1977	1.145 (1.067 −1.228)	<0.001		10504	725	0.932 (0.839-1.034)	0.185
50-64	11489	2263	1.057 (0.988-1.131)	0.110		12827	910	0.958 (0.867-1.057)	0.391
≥65	13051	1648	0.678 (0.630-0.728)	<0.001		13908	768	0.745 (0.673-0.826)	<0.001
**Marital status**									
Single	10331	1989	Ref			11442	878	Ref	
Married/common law	23796	3918	0.855 (0.807-0.907)	<0.001		26049	1622	0.811 (0.745-0.883)	<0.001
Widowed/divorced/separated	9151	1738	0.986 (0.920-1.058)	0.704		10191	674	0.862 (0.777-0.956)	0.005
**Income**									
Less than $20,000	574	131	Ref			646	58	Ref	
$20,000 to $59,999	23854	4719	0.867 (0.718-1.055)	0.145		26495	2038	0.857 (0.659-1.137)	0.266
$60,000 to $79,999	12525	1872	0.655 (0.540-0.800)	<0.001		13627	729	0.612 (0.467-0.817)	<0.001
$80,000 or more	6355	926	0.638 (0.523-0.784)	<0.001		6936	337	0.541 (0.408-0.730)	<0.001
**Education**									
Less than secondary school	6132	1317	Ref			6856	597	Ref	
Secondary school graduation	9543	1882	0.918 (0.850-0.992)	0.031		10652	772	0.832 (0.745-0.930)	0.001
Post-secondary certificate/univ degree	27118	4348	0.747 (0.698-0.799)	<0.001		29625	1778	0.689 (0.626-0.759)	<0.001
**General Health**									
Good	38935	5514	Ref			42029	2364	Ref	
Poor	4388	2144	3.455 (3.253-3.659)	<0.001		5701	815	2.542 (2.339-2.765)	<0.001
**Mental Health**									
Good	41062	6148	Ref			44533	2618	Ref	
Poor	2242	1496	4.457 (4.152-4.783)	<0.001		3175	557	2.984 (2.705-3.292)	<0.001
**BMI**									
Normal Weight	17938	2816	Ref			19522	1193	Ref	
Underweight	830	178	1.366 (1.153-1.610)	<0.001		938	72	1.256 (0.974-1.596)	0.070
Overweight	14731	2339	1.011 (0.953-1.073)	0.706		16040	1023	1.044 (0.958-1.137)	0.331
Obese—Class I, II, III	8475	2024	1.521 (1.429-1.620)	<0.001		9722	776	1.306 (1.189-1.434)	<0.001
**Alcohol Drinking**									
No drinking in the last year	7716	1614	Ref			8639	680	Ref	
Occasional	6520	1481	1.085 (1.005-1.174)	0.038		7413	568	0.973 (0.867-1.093)	0.648
Regular	28971	4540	0.749 (0.704-0.797)	<0.001		31555	1921	0.773 (0.706-0.847)	<0.001
**Smoking**									
Never	21809	3355	Ref			23720	1389	Ref	
Current	7583	1969	1.688 (1.587-1.795)	<0.001		8736	796	1.556 (1.421-1.703)	<0.001
Former	13858	2329	1.092 (1.032-1.165)	0.002		15197	994	1.117 (1.027-1.215)	0.010

## Results

Weighted prevalence estimates of sleep disturbances varied across sociodemographic, health, and behavioral characteristics, as illustrated in the heat map (Fig 2). Female participants reported rates of inadequate sleep (41%), insomnia (22%), and unrefreshing sleep (16%), while the corresponding rates among males were 44%, 14%, and 13%, respectively. Regarding age, individuals aged 35–49 and 50–64 years had the highest weighted prevalence of inadequate sleep and insomnia, while those aged 65 years and older showed the lowest rates across all sleep outcomes. Participants with lower educational attainment (less than secondary school graduation) exhibited the highest weighted prevalence of inadequate sleep (45%), insomnia (22%), unrefreshing sleep (18%), and difficulty staying awake (8%). Participants who were separated, divorced, or widowed exhibited the highest weighted prevalence of inadequate sleep (45%), insomnia (22%), and unrefreshing sleep (16%). Respondents with an annual income of less than $20,000 had the highest prevalence of inadequate sleep (45%), insomnia (21%), unrefreshing sleep (17%). Marked disparities were observed by self-reported health status; individuals reporting poor general health had substantially higher rates of inadequate sleep (53%), insomnia (41%), unrefreshing sleep (33%), and difficulty staying awake (12%). Similarly, participants with poor mental health exhibited elevated prevalence across all sleep outcomes: inadequate sleep (55%), particularly insomnia (45%), unrefreshing sleep (40%), and difficulty staying awake (15%). Obesity and occasional smoking were also associated with higher prevalence of sleep disturbances, whereas regular alcohol consumption was linked to lower rates across most sleep measures. Among smoking status categories, former smokers had the highest weighted prevalence of inadequate sleep (47%), insomnia (23%), unrefreshing sleep (20%), and difficulty staying awake (8%).

**Fig 2:**
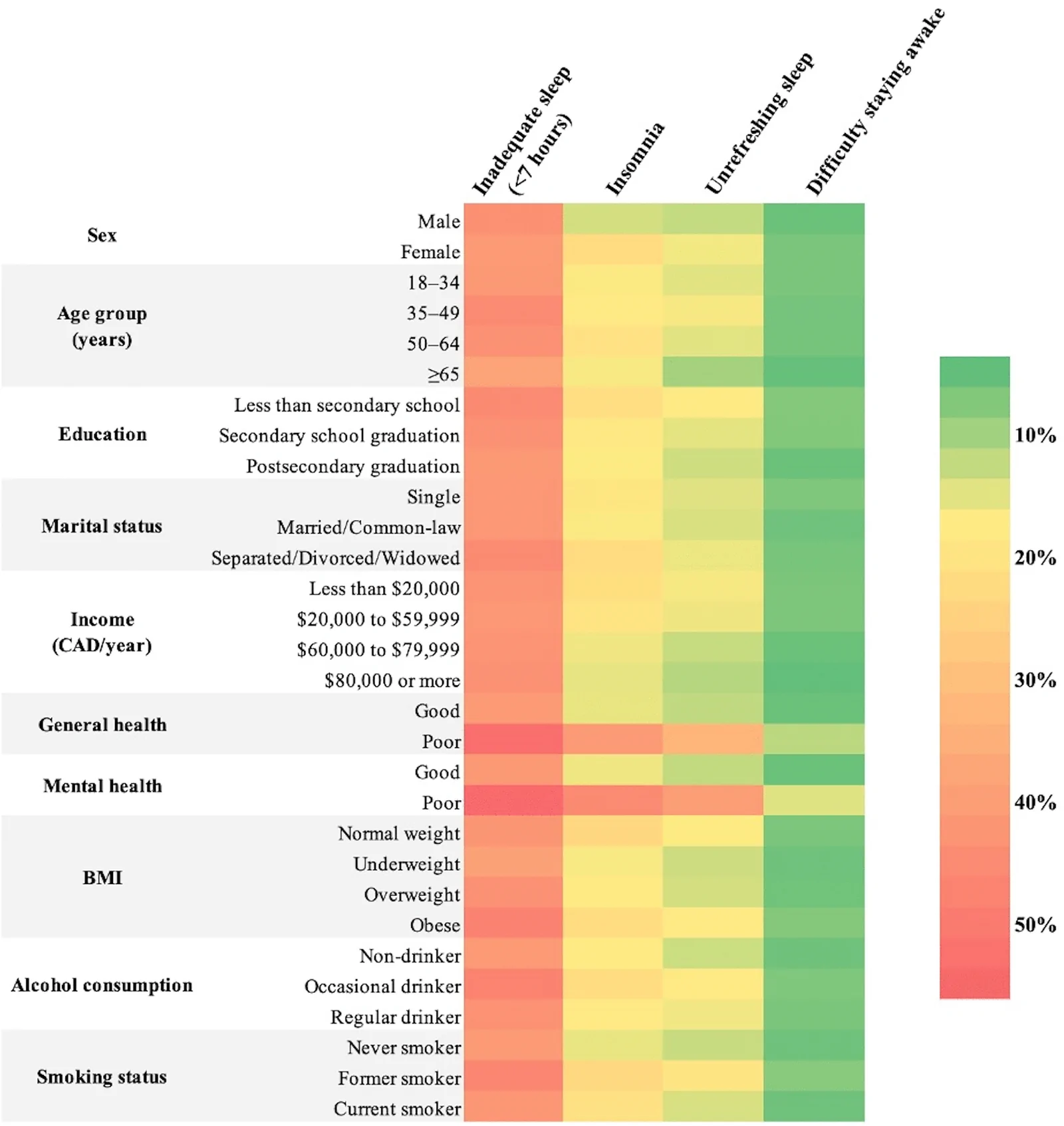
Heatmap showing the weighted percentage of four sleep disturbances (short sleep duration, insomnia, unrefreshing sleep, and difficulty staying awake) by sociodemographic, health‑related, and lifestyle factors among Canadian adults (CCHS 2017–2018). Color intensity indicates the percentage of poor outcomes (green = low, red = high).

Univariate logistic regression was used to examine associations between demographic and lifestyle factors with four sleep-related outcomes (Table 1). Our results showed that females were less likely to report short sleep duration (OR 0.861) compared to males. However, females were more likely to report insomnia (OR 1.646), unrefreshing sleep (OR 1.309), and difficulty staying awake (OR 1.161) compared to males. Compared to participants aged 18–34 years, those aged 35–49 years (OR 1.151) and 50–64 years (OR 1.184) were more likely to report short sleep duration, while those ≥65 years were less likely to report short sleep duration (OR 0.931). The participants aged 35–49 years and 50–64 years were more likely to report insomnia (OR 1.094 and OR 1.325, respectively) compared to those aged 18–34 years. Individuals who were married/common-law were less likely to report short sleep duration (OR 0.913) and insomnia (OR 0.817) compared to single individuals. Participants with a post-secondary certificate/university degree were less likely to report short sleep duration (OR 0.860), insomnia (OR 0.803), unrefreshing sleep (OR 0.747), and difficulty staying awake (OR 0.689) compared to those with less than secondary school graduation. Participants who reported their perceived general health as poor were more likely to report short sleep duration (OR 1.651), insomnia (OR 3.368), unrefreshing sleep (OR 3.455), and difficulty staying awake (OR 2.542) compared with participants who reported good general health. Similarly, individuals who reported their perceived mental health as poor were more likely to report short sleep duration (OR 1.679), insomnia (OR 4.228), unrefreshing sleep (OR 4.457), and difficulty staying awake (OR 2.984) compared with participants who reported good mental health. Participants with obesity were more likely to report short sleep duration (OR 1.343), insomnia (OR 1.383), unrefreshing sleep (OR 1.521), and difficulty staying awake (OR 1.306) compared to participants with normal BMI. Regular alcohol drinkers were less likely to report short sleep duration (OR 0.875), insomnia (OR 0.927), unrefreshing sleep (OR 0.749), and difficulty staying awake (OR 0.773) compared to those who never drink. Current smokers were more likely to report short sleep duration (1.336), insomnia (1.665), unrefreshing sleep (OR 1.688), and difficulty staying awake (OR 1.556) compared to non-smoker participants.

Table 2 shows the association between oral health problems and sleep duration and quality. In the fully adjusted model (Model 4), all oral health indicators were significantly associated with inadequate sleep duration (short sleep <7 hours). Individuals with poor oral health were 1.623 times more likely to report short sleep compared to those with good oral health. In addition, individuals with oral pain (AOR: 1.309), bleeding gums (AOR: 1.148), and dry mouth (AOR: 1.438) were more likely to report inadequate sleep duration (<7 hours) compared to those without these conditions. Participants with poor oral health were (AOR: 1.508) more likely to report insomnia compared to those with good oral health. Moreover, participants with oral pain (AOR: 1.826), bleeding gums (AOR: 1.386), and dry mouth (AOR: 1.904) were more likely to report insomnia compared to participants without these issues. In addition, individuals with poor oral health were more likely to report unrefreshing sleep (AOR: 1.768) compared to those with good oral health. Participants with oral pain and those with dry mouth were more likely to report unrefreshing sleep compared to those without oral pain or without dry mouth (AOR: 1.937 and AOR: 1.808, respectively). Participants with oral pain (AOR: 1.701) and dry mouth (AOR: 1.720) were more likely to report difficulty staying awake compared to those without these conditions. After Benjamini–Hochberg false discovery rate (FDR) correction, all 13 associations that were statistically significant in Model 4 remained significant, while the three nonsignificant associations remained nonsignificant (Supplementary Table 2).

**Table 2:** Associations between oral health (poor oral health, oral pain, bleeding gums, and dry mouth) and sleep outcomes (short sleep duration, insomnia, unrefreshing sleep, and difficulty staying awake) among Canadian adults aged 18 years and older (Canadian Community Health Survey, 2017–2018). Data are presented as frequencies, odds ratios (OR) for Model 1: represents the unadjusted (crude) association; and Model 4: represents the association adjusted for sex, age, education, BMI, smoking status, alcohol drinking, income, marital status, perceived General Health, and perceived mental health. (Partially adjusted models (Models 2 and 3) are shown in Supplementary Material 1). All analyses were weighted. Statistical significance was set at p < 0.05. Ref = reference category, N = number of respondents.

	Sleep duration					
	Adequate duration (≥7) (N)	Inadequate duration (<7) (N)	Model 1 OR (95% CI)	P-value	Model 4 AOR (95% CI)	P-value
**Oral Health**						
Good	12976	7491	Ref		Ref	
Poor	1044	1062	1.879 (1.631–2.165)	<0.001	1.623 (1.392-1.891)	<0.001
**Oral Pain**						
No	12584	7410	Ref			
Yes	1432	1142	1.345 (1.175–1.541)	<0.001	1.309 (1.134-1.512)	<0.001
**Bleeding gums**						
No	9075	5241	Ref			
Yes	3141	2172	1.178 (1.066–1.302)	<0.001	1.148 (1.031-1.280)	0.012
**Dry mouth**						
No	11042	6252	Ref			
Yes	2961	2291	1.489 (1.345–1.648)	<0.001	1.438 (1.288-1.605)	<0.001
	**Insomnia**					
	No	Yes	Model 1		Model 4	
	(N)	(N)	OR (95% CI)	P-value	AOR (95% CI)	P-value
**Oral Health**						
Good	17098	3401	Ref		Ref	
Poor	1481	629	2.075 (1.764–2.440)	<0.001	1.508 (1.252-1.815)	<0.001
**Oral Pain**						
No	16771	3260	Ref		Ref	
Yes	1806	770	2.236 (1.939–2.579)	<0.001	1.826 (1.555-2.143)	<0.001
**Bleeding gums**						
No	12020	2323	Ref		Ref	
Yes	4131	1182	1.421 (1.263–1.599)	<0.001	1.386 (1.217-1.577)	<0.001
**Dry mouth**						
No	14769	2545	Ref		Ref	
Yes	3791	1482	2.405 (2.145–2.696)	<0.001	1.904 (1.676-2.161)	<0.001
	**Unrefreshing Sleep**					
	Yes	No	Model 1		Model 4	
	(N)	(N)	OR (95% CI)	P-value	AOR (95% CI)	P-value
**Oral Health**						
Good	2032	18409	Ref		Ref	
Poor	474	1635	2.819 (2.355-3.375)	<0.001	1.768 (1.450-2.157)	<0.001
**Oral Pain**						
No	1983	17985	Ref		Ref	
Yes	521	2056	2.449 (2.050-2.926)	<0.001	1.937 (1.597-2.350)	<0.001
**Bleeding gums**						
No	1415	12893	Ref		Ref	
Yes	725	4584	1.415 (1.216-1.647)	<0.001	1.159 (0.985-1.393)	0.076
**Dry mouth**						
No	1548	15725	Ref		Ref	
Yes	953	4304	2.317 (2.005-2.677)	<0.001	1.808 (1.528-2.140)	<0.001
	**Difficult to stay awake**					
	No	Yes	Model 1		Model 4	
	(N)	(N)	OR (95% CI)	P-value	AOR (95% CI)	P-value
**Oral Health**						
Good	19075	1368	Ref		Ref	
Poor	1851	252	1.586 (1.277–1.970)	<0.001	1.243 (0.975-1.585)	0.079
**Oral Pain**						
No	18659	1311	Ref		Ref	
Yes	2266	308	1.914 (1.561–2.346)	<0.001	1.701 (1.367-2.116)	<0.001
**Bleeding gums**						
No	13385	913	Ref		Ref	
Yes	4833	474	1.255 (1.050–1.499)	0.012	1.196 (0.992-1.442)	0.061
**Dry mouth**						
No	16238	1031	Ref		Ref	
Yes	4668	589	1.947 (1.630–2.326)	<0.001	1.720 (1.413-2.094)	<0.001

## Discussion

This cross-sectional study provides comprehensive evidence that oral health problems are consistently associated with multiple dimensions of sleep disturbance in a representative sample of Canadian adults. Using nationally representative data from the 2017–2018 CCHS, we found that poor oral health, oral pain, bleeding gums, and dry mouth were significantly associated with short sleep duration, insomnia symptoms, unrefreshing sleep, and difficulty staying awake, even after adjusting for a broad range of sociodemographic, health status, and behavioral confounders. Although these sleep measures are interrelated and may co-occur clinically, they capture distinct dimensions of sleep health, quantity, quality, and daytime functioning, and their differing patterns of association with the oral health indicators in this study indicate that they should not be considered interchangeable [1]. To our knowledge, this is one of the first studies to examine the associations between four distinct oral health indicators and four separate sleep outcomes in a large nationally representative adult population. A previous study by members of our group using the same CCHS cycle examined associations between lifestyle and health characteristics, including sleep and oral health, among Canadians with arthritis [13]. However, it did not specifically examine the association between oral health indicators and sleep outcomes in the general adult population.

Our findings are broadly consistent with and extend the international evidence base. Poor oral health, often assessed as a composite of symptoms and functional limitations, has been shown to reflect underlying clinical conditions and is associated with quality of life [8]. In our analysis, poor oral health was consistently associated with sleep disturbances. Sleep disorders have been significantly associated with adverse oral health outcomes in US adults [12]. Our study is complementary in demonstrating that the association persists when oral health is positioned as the exposure, supporting a bidirectional relationship. A significant association between short sleep duration and dental caries was identified in a nationally representative U.S. sample, suggesting that the relationship between sleep and oral health extends to specific clinical pathologies beyond self-reported measures [21]. Moreover, adults with poor oral health were found to be more likely to experience shorter sleep duration, reinforcing the population-level relevance of this association [22]. The association between oral health and sleep problems, examined in the current study, offers a comprehensive view of the oral health–sleep interface in Canadian adults.

Our analyses revealed sociodemographic patterns influence sleep outcomes. Females reported higher rates of insomnia, consistent with well-documented sex differences in sleep disturbance prevalence [23]. Individuals aged 35–49 and 50–64 years had the highest prevalence of inadequate sleep, while those aged ≥65 years had the lowest rates, possibly reflecting retirement-related improvements in sleep schedules. Poor general health and poor mental health had the strongest associations with sleep disturbances, consistent with extensive literature documenting the interrelationship between chronic disease burden, mental health, and sleep [2,3]. Current smoking was associated with higher odds of all adverse sleep outcomes, consistent with nicotine’s stimulant effects and its known associations with sleep disruption [24].

Our analysis revealed oral pain exhibited significant associations with sleep disturbance, including short sleep duration, insomnia, and difficulty staying awake. Importantly, the associations between oral health indicators and sleep outcomes persisted after adjustment for sociodemographic, health status, and behavioral confounders. These findings are consistent with established evidence on the bidirectional relationship between pain and sleep. Chronic pain is among the most common causes of insomnia, and reciprocally, sleep disruption amplifies pain sensitivity through central sensitization and altered descending inhibitory pathways [7,25,26]. In the orofacial domain, a circular mutually detrimental interaction has been described whereby chronic orofacial pain disrupts sleep architecture, and fragmented sleep subsequently lowers pain thresholds and intensifies pain perception [7]. Similarly, the interaction between sleep and orofacial pain has direct relevance for dental practice, as unmanaged dental pain may contribute to insomnia, reduced restorative sleep, and daytime fatigue [27]. Moreover, sleep disturbances are associated with both pain intensity and pain-related functional interference in patients with orofacial conditions [28]. Our findings extend this evidence by demonstrating that oral pain is associated with impairment across multiple sleep dimensions, beyond sleep onset difficulty alone. The association between oral pain and excessive daytime sleepiness is notable, given its implications for occupational safety, driving performance, and overall productivity [2], suggesting that oral pain affects daytime functioning and quality of life beyond sleep disturbance.

Dry mouth exhibited a strong association with insomnia, difficulty staying awake, short sleep duration, and reduced refreshing sleep. These findings are consistent with the clinical observation that xerostomia is a particularly disruptive condition during sleep. Patients with xerostomia had significantly poorer sleep quality and greater daytime sleepiness, as repeated nocturnal awakenings due to oral dryness, discomfort, and the need to drink water disrupt sleep continuity and reduce restorative slow-wave sleep [10]. A recent review emphasized xerostomia as a key driver of sleep disruption with cascading effects on nutrition, quality of life, and well‑being [29]. An equally plausible direction of association is that dry mouth may be a marker of sleep-disordered breathing rather than a contributor to poor sleep: nocturnal mouth breathing secondary to obstructive sleep apnea (OSA), allergic rhinitis, or nasal obstruction directly produces xerostomia, and dry mouth upon awakening is a recognized correlate of OSA severity [30,31]. The strong associations we observed between dry mouth and both insomnia symptoms and difficulty staying awake are consistent with this reverse-direction interpretation. Our findings are consistent with xerostomia sleep relationship and suggest that managing xerostomia may serve as a useful marker in sleep health assessment and optimization strategies.

A unifying neurophysiological framework may link oral pain and dry mouth findings through trigeminal afferent pathways. Trigeminal nociceptive input projects to the ascending reticular activating system and locus coeruleus, providing a neuroanatomical route by which orofacial nociception can fragment sleep architecture [7,32]. Reciprocally, sleep fragmentation lowers pain thresholds via impaired descending inhibitory control [33], suggesting a bidirectional association between orofacial pain and sleep disturbance, although our cross-sectional data cannot resolve directionality. Notably, the nasal mucosa is richly innervated by trigeminal chemosensory and airflow receptors that contribute to arousal regulation, so the nocturnal mouth breathing that produces dry mouth may concurrently alter trigeminal signaling to cortical arousal networks, leading to arousal and sleep fragmentation, placing xerostomia within the same framework [34,35]. This interpretation is consistent with our finding that both symptoms were strongly associated with poor sleep outcomes and may provide a biologically plausible explanation for their co‑occurrence.

Short sleep duration, poor sleep quality, and insomnia complaints are associated with increased risk of periodontal disease through shared inflammatory pathways [6]. Aligning with this evidence, our analysis found that bleeding gums, a clinical indicator of gingival inflammation and potential periodontal disease, showed significant associations with short sleep duration and insomnia outcomes. Periodontal pathogens and their products enter the systemic circulation, elevating C-reactive protein and pro-inflammatory cytokines (e.g., IL-6, TNF-α) that are independently implicated in sleep dysregulation [36,37]. The relatively weaker magnitude of associations for bleeding gums compared to oral pain and dry mouth may reflect the fact that gingival bleeding is often painless and may not directly impair sleep through discomfort or nocturnal awakening.

### Clinical and public health implications

Our findings have several interconnected implications. Dental professionals should routinely assess sleep quality in patients with oral pain, xerostomia, or poor oral health, while primary care and sleep specialists should include oral health evaluations for patients with sleep complaints. Given the association between xerostomia and insomnia, clinicians may consider oral health, including dry mouth, when evaluating patients with sleep complaints, particularly older adults with polypharmacy. Dry mouth complaints, especially upon awakening, should raise suspicion of undiagnosed sleep-disordered breathing and prompt referral, rather than symptomatic management alone. Similarly, oral pain assessment may also be relevant alongside standard sleep evaluation. Whether targeted oral health interventions improve sleep outcomes causally remains to be tested in longitudinal or interventional studies. Public health initiatives, including the Canadian Dental Care Plan, should address the interconnected nature of sleep and oral health, particularly in populations with limited dental access. Finally, stronger interprofessional collaboration between dental and sleep medicine professionals is needed to address the overlapping determinants and consequences of poor oral and sleep health.

### Limitations

Although our study used a large, nationally representative sample (CCHS), several limitations merit mention. First, the cross-sectional design precludes causal inference. Although bidirectional pathways between oral and sleep health have been proposed in the literature, the present cross-sectional associations cannot establish these pathways, and future longitudinal studies should test them directly. Second, all measures were self-reported, introducing the possibility of recall bias, reporting bias, and misclassification. Self-reported oral health may not fully reflect clinically diagnosed oral conditions, while self-reported sleep measures may not perfectly correspond to objectively assessed sleep parameters. Consequently, the observed associations should be interpreted with appropriate caution. Moreover, this study could not account for potential confounding by medication use or obstructive sleep apnea because sufficiently detailed information on these variables was not available in the CCHS Public Use Microdata File. As these factors may influence both oral health, particularly xerostomia, and sleep outcomes, residual confounding cannot be completely excluded.

### Future directions

Several avenues for future research emerge from our findings. Longitudinal studies are needed to establish temporal relationships and bidirectionality. Mechanistic research examining inflammatory biomarkers, salivary cortisol, and polysomnographic sleep architecture would elucidate biological pathways. Randomized controlled trials evaluating the effects of dental treatments, such as pain management, periodontal therapy, and xerostomia interventions, on sleep outcomes would provide the strongest evidence for integrated care. Studies should examine obstructive sleep apnea as a potential mediator, particularly for xerostomia. Moreover, future studies should incorporate clinical oral examinations, salivary flow measurements, and objective sleep assessments (actigraphy, polysomnography) to complement self‑reported data. In addition, because reduced salivary flow has been associated with alterations in the oral microbiome, including a shift toward a dysbiotic, pro-inflammatory state [38], future studies combining objective salivary flow measurements with microbiome profiling may help clarify a potential pathway linking dry mouth and gingival inflammation with sleep outcomes. Importantly, mechanistic studies combining polysomnography, quantitative trigeminal sensory testing, and inflammatory and salivary biomarkers would be particularly valuable to evaluate the relationship between trigeminal nociception, sleep fragmentation, descending inhibition, and oral symptoms, thereby identifying therapeutic targets.

## Conclusion

This large, nationally representative study demonstrates that oral health problems, including poor oral health, oral pain, bleeding gums, and dry mouth, are associated with multiple dimensions of sleep disturbance (short sleep duration, insomnia, daytime sleepiness, and unrefreshing sleep) in Canadian adults. Dry mouth and oral pain showed the strongest associations with insomnia and daytime sleepiness, while dry mouthand oral pain were most strongly associated with unrefreshing sleep. These findings underscore the interconnectedness of oral and sleep health and highlight the need for integrated clinical approaches, interprofessional collaboration, and public health strategies that address both dimensions simultaneously. In addition, incorporating oral health checks into sleep medicine and assessing sleep quality in dental settings may become a valuable aspect of comprehensive, patient-focused care.
